# 

**Published:** 1899-08

**Authors:** 


### Communicated.

WHY SHOULD NEW JERSEY COMPEL DENTISTS TO REGISTER ANNUALLY.—This is a question of some importance to the dental profession of New Jersey, and in view of the fact that this State is of no account financially to that profession, and it is not recognized in any of the public institutions, and is stringent and unjust in its legislation, we assume the ground, that in the light of the above facts, that this State has no right whatever for such tyrannical, unjust and unconstitutional enactments.

In the entire state of New Jersey some half dozen men, known as the New Jersey State Board of Registration and Examination in Dentistry, are recognized by the law. It is a question if the gentlemen constituting the board receive a single cent from the State Treasury.

If the dental profession was recognized in all the state and public institutions, this State to a certain extent should look after its members, but as a matter of absolute fact, the dental profession is ignored.

How different it is with the medical profession. The State has its Board of Health, etc. Every city, country, township and even small towns have their public physicians, paid out of the public treasury for their services. A physician living less than six miles from the writer's office, has been paid in less than fifteen years, more than three thousand dollars for services rendered the poor and indignent in his township.

The writer has performed dental operations for the poor for upwards of a quarter of a century, and never received a single dollar for his services, either from the poor, "the State's wards," or from the state, township or county. Hence I dispute the right of New Jersey to hold the reins over the dental prefession. It is unjust, cruel, disgraceful. tyrannical, degrading and fraudulent.

Right here we would say, that if the profession of dentistry in New Jersery had stood by us in our opposition to the these laws, they would never have been enacted. Almost unaided the writer had a proposed dental law "strangled" in committee of the Senate, but it passed the succeding year because of no opposition.

United States legislation and Josiah Bacon, representing the Goodyear Dental Vulcanite Company, had everything their own way, and acts often vile, cowardly and sometimes serreptious, will meet with a well deserved fate in some of the American States, if American manhood is not dead.

There are too many laws in America, many of them conflicting. Americans are governed too much. The governing of mankind is at best a necessary evil. "That country is governed best, that is governed least." The very stringency of some laws betray their weakness and defeat their object.

DR. EDWARD H. BOWNE,
Kingston, N. J., April, 1899.

---

### COLORADO STATE DENTAL ASSOCIATION.

At the thirteenth Annual Meeting of the Colorada State Dental Association, held in Denver, June 13th, 14th and 15th, 1899, the following officers were elected:

President, A. C. Watson, Denver; 1st Vice-President, J. N. Chipley, Pueblo; 2nd Vice-President. Mary A. Bradner, Denver; Cor. Secretary, Florence S. Green, Denver; Record. Secretary, L. S. Gilbert, Denver; Treasnrer, William Smedley, Denver.

FLORENCE S. GREEN, Cor. Sec'y.
207 Mack Block, Denver.

---

### Editorial, Etc.

WILL FIGHT THE DECISION.—DENTISTS TO OPPOSE THE INTERNATIONAL TOOTH CROWN COMPANY.—Dr. J. N. Crouse, president of the Dental Protective Association, which is in session at Niagara Falls, N. Y., said that a meeting would be held Friday morning to take action upon the decision rendered in New York by Judge Townsend in favor of the International Tooth Crown Company, as against Dr. James Orr Kyle, a dentist.

The decision sustains the validity of the company's patents on all operations known as "tooth crowns" and "bridge work," and allows the company to collect royalties on all infringements. It is said that claims conservatively estimated at $10,000,000 are affected.

Dr. Crouse says that hundreds of dentists will join the Protective Association, and that the decision will be fought.

### To Readers and Correspondents.

Communications intended for publication, and exchange journals and papers, should be addressed to the Editor of The American Journal of Dental Science, No. 845 N. Eutaw St. Baltimore, Md. All letters relating to the business of the Journal, or containing cash remittances, to be directed to Snowden & Cowman Mfg. Co., 9 W. Fayette Street, Baltimore, Md. Articles for publication should be received by the Editor at least two weeks previous to the first of the month on which the number in which it is designed for them to appear is issued.

☞ The American Journal of Dental Science will contain fifty pages of reading matter in each number, making a volume of about Six Hundred pages,

EXCLUSIVE OF ADVERTISEMENTS.

